# PBRM-1/PBAF-regulated genes in a multipotent progenitor in *Caenorhabditis elegans*

**DOI:** 10.1093/g3journal/jkad297

**Published:** 2023-12-27

**Authors:** Laura D Mathies, Andrew C Kim, Evan M Soukup, Alan’da E Thomas, Jill C Bettinger

**Affiliations:** Department of Pharmacology and Toxicology, Virginia Commonwealth University, Richmond, VA 23298, USA; Department of Pharmacology and Toxicology, Virginia Commonwealth University, Richmond, VA 23298, USA; Department of Pharmacology and Toxicology, Virginia Commonwealth University, Richmond, VA 23298, USA; Department of Pharmacology and Toxicology, Virginia Commonwealth University, Richmond, VA 23298, USA; Department of Pharmacology and Toxicology, Virginia Commonwealth University, Richmond, VA 23298, USA

**Keywords:** SGP, hmc, multipotent progenitor, *C. elegans*, *pbrm-1*, PBAF, muscle, differential gene expression, SWI/SNF chromatin remodeling

## Abstract

The *Caenorhabditis elegans* somatic gonadal precursors (SGPs) are multipotent progenitors that generate all somatic cells of the adult reproductive system. The 2 SGPs originate in the mesodermal layer and are born through a division that produces one SGP and one head mesodermal cell (hmc). One hmc terminally differentiates, and the other dies by programmed cell death. The polybromo-associated BAF (PBAF) chromatin remodeling complex promotes the multipotent SGP fate. The complete loss of PBAF causes lethality, so we used a combination of Cre/lox recombination and GFP nanobody-directed protein degradation to eliminate PBRM-1, the signature subunit of the PBAF complex, from 83 mesodermal cells, including SGPs, body muscles, and the hmc. We used RNA sequencing to identify genes acting downstream of PBAF in these cells and identified 1,955 transcripts that were significantly differentially expressed between *pbrm-1(−)* and *pbrm-1(+)* in the mesoderm of L1 larvae. We found that genes involved in muscle cell function were overrepresented; most of these genes had lower expression in the absence of PBRM-1, suggesting that PBAF promotes muscle differentiation. Among the differentially expressed genes were 125 that are normally expressed at higher levels in SGP vs hmc and positively regulated by *pbrm-1* and 53 that are normally expressed at higher levels in hmc vs SGP and are negatively regulated by *pbrm-1;* these are candidate regulators of the SGP/hmc fate decision. We validated one candidate gene using a fluorescent reporter; the *hsp-12.3* reporter was derepressed in SGPs in *pbrm-*1 mutants, suggesting that *hsp-12.3* expression is normally repressed by *pbrm-1* in SGPs.

## Introduction

SWItching defective/sucrose nonfermenting (SWI/SNF) chromatin remodeling complexes are multiprotein assemblies that regulate gene expression by altering chromatin structure (Clapier and Cairns 2009). Two major classes of SWI/SNF complexes are BRM/BRG-associated factor (BAF) and polybromo-associated BAF (PBAF; reviewed in Wu *et al.* 2009). In mammals, molecularly distinct BAF complexes are found in pluripotent embryonic stem cells (esBAF), multipotent neural progenitors (npBAF), and differentiated neurons (nBAF; Lessard *et al.* 2007; Ho *et al.* 2009; Lessard and Crabtree 2010). SWI/SNF subunits facilitate the reprogramming of differentiated cells into pluripotent stem cells (Singhal *et al.* 2010), underscoring the importance of SWI/SNF complexes in the regulation of cellular potential.

The *Caenorhabditis elegans* somatic gonadal precursors (SGPs) are multipotent progenitors that generate all 143 cells of the somatic gonad. The 2 SGPs, Z1 and Z4, are born from cell divisions that produce one SGP and one head mesodermal cell (hmc; Sulston *et al.* 1983). After their births, the SGPs migrate posteriorly and coalesce with the primordial germ cells to form the gonad primordium. Each SGP generates 1 of the 2 U-shaped arms of the adult gonad (Kimble and Hirsh 1979). The hmcs migrate anteriorly where one cell dies by programmed cell death and the other differentiates as the single hmc. We previously defined the transcriptomes of sorted SGPs and hmcs and found that they had numerous transcriptional differences: ∼3,000 genes had higher expression in SGP than hmc (SGP-biased), and a similar number had higher expression in hmc than SGP (hmc-biased; Mathies *et al.* 2019). Thus, sister cells that were produced by a single cell division have very different gene expression profiles consistent with their different cellular potentials (terminally differentiated vs multipotent).

Little is known about the regulation of the SGP/hmc fate decision. Mutations in 4 genes, the conserved mesoderm regulator *hnd-1/*dHand, and 3 genes encoding subunits of the PBAF chromatin remodeling complex play a role in regulating this cell fate decision (Large and Mathies 2014). Animals carrying mutations in any of the genes have 2 incompletely penetrant phenotypes: (1) SGPs can be absent from the gonad primordium resulting in adults with missing gonad arms and (2) SGPs can have gene expression patterns characteristic of both SGP and hmc. Both phenotypes can be explained by the partial transformation of SGPs into hmcs and suggest that these genes are important for distinguishing multipotent SGPs from their differentiated hmc sisters. The involvement of 3 PBAF genes, including genes encoding the signature subunit PBRM-1*/*Polybromo and the core ATPase SWSN-4, strongly suggests that *C. elegans* PBAF regulates cellular potential, as has been shown for mammalian BAF complexes.

Here, we used a combination of Cre/lox recombination and GFP nanobody-directed protein degradation to deplete *pbrm-1* mRNA and protein from mesodermal tissues, including SGPs. The mesodermal inactivation of *pbrm-1* resulted in a strong loss-of-function phenotype in the somatic gonad, without the high degree of lethality associated with null alleles of the gene. Using this conditional inactivation strategy, we identified 1,955 genes that were differentially expressed in the mesoderm between *pbrm-1(*−*)* and *pbrm-1(+)*. Genes implicated in muscle function were overrepresented among the differentially expressed genes (DEGs), suggesting a role for PBRM-1 in muscle cell differentiation. To find genes that may be important for the SGP/hmc fate decision, we utilized an existing gene expression dataset from sorted SGPs and hmcs (Mathies *et al.* 2019) to identify 178 candidate mediators of the *pbrm-1* effect on the SGP/hmc fate decision. We used a fluorescent reporter to validate one candidate gene, *hsp-12.3,* which had hmc-biased expression in wild-type animals and became derepressed in SGPs of *pbrm-1* mutants, indicating that we can identify *pbrm-1*–regulated genes in SGPs using this dataset.

## Materials and methods

### Strains

*C. elegans* strains were cultured as described previously (Brenner 1974; Wood 1988). All strains were grown at 20°C unless otherwise specified and were derived from the Bristol strain N2. Strains were obtained from the *Caenorhabditis* Genetics Center or were generated as described below. A complete list of strains is included in Supplemental material online (Supplementary File 1). The following strains were generated for this study:

RA650 *pbrm-1(rd29[pbrm-1::GFP::3xFlag])*

RA661 *pbrm-1(rd31[pbrm-1::GFP(flox)])*

RA663 *pbrm-1(rd31[pbrm-1::GFP(flox)]); rdIs67[hnd-1p::Cre]*

RA678 *pbrm-1(rd31[pbrm-1::GFP(flox)]); rdIs74[hnd-1p::GFP-nanobody::ZIF-1::Cre]*

RA685 *pbrm-1(rd31[pbrm-1::GFP(flox)]); rdIs79[hnd-1p::ZIF-1]*

### Generation of loxP-flanked *pbrm-1::GFP*

**Fig. 1.**
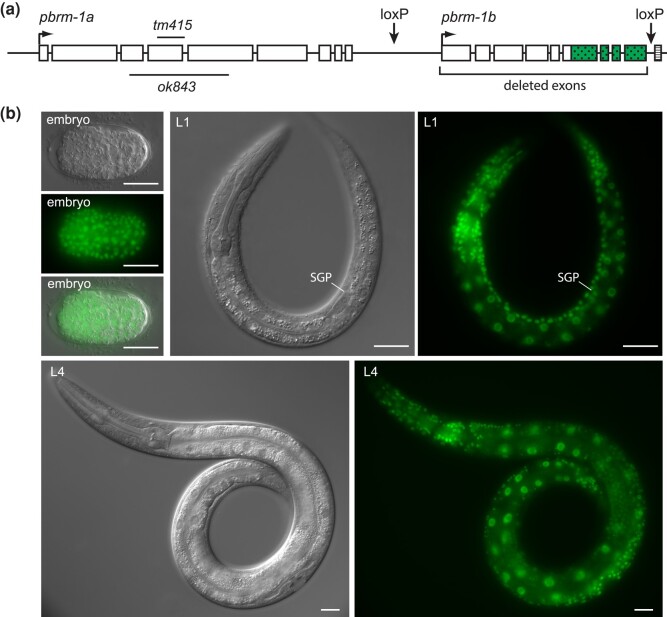
Generation of a GFP-tagged loxP-flanked *pbrm-1* allele. a) The engineered *pbrm-1* locus contains GFP-coding sequences (stippled) immediately downstream of the last *pbrm-1* coding exon, followed by a 3× FLAG tag (striped). One loxP site remains between GFP and 3× FLAG following removal of the self-excising cassette. An additional loxP site was inserted upstream of the start of *pbrm-1b.* Cre-mediated recombination removes the last 6 exons of *pbrm-1* and all GFP-encoding exons; the deleted locus is expected to produce a strong loss of function because it removes all common *pbrm-1* exons. b) PBRM-1::GFP is found in the nucleus of many and perhaps all cells across development, from embryogenesis through adulthood. Images shown are embryos, L1 larvae, and L4 larvae.

GFP coding sequences were inserted just before the *pbrm-1* stop codon using Clustered Regularly Interspaced Short Palindromic Repeats (CRISPR)/Cas9 genome editing. A repair plasmid was generated by cloning sequences flanking the intended insertion site (homology arms) into pDD282 (Addgene #66823) digested with AvrII and SpeI. The left homology arm was amplified by PCR using primers RA1386 and RA1387, and the right homology arm was synthesized as a gblock gene fragment (IDT, Skokie, IL, USA). The guide sequence was cloned into PU6::unc-119_sgRNA (Addgene #46169; Friedland *et al.* 2013) using the Q5 site directed mutagenesis kit (NEB, Ipswich, MA, USA) and primers RA1318 and RA1063. Insertions were created following a published protocol (Dickinson *et al.* 2015). Briefly, repair (10 ng/μl), P*eft-3::*Cas9 (50 ng/μl), and guide (50 ng/μl) plasmids were injected into N2 worms with fluorescent coinjection markers. Insertions were selected using a combination of hygromycin resistance and the *sqt-1(d)* roller phenotype; both markers are contained within the self-excising selection cassette of pDD282. The selection cassette was removed by heat shock to create RA650, which retains one loxP site in the final intron between GFP and 3xFlag (Fig. 1a). A second loxP site was inserted 721 bp upstream of the start of *pbrm-1b* using CRISPR/Cas9. The guide sequence was cloned into PU6::unc-119_sgRNA (Addgene #46169; Friedland *et al.* 2013) using primers RA1063 and RA1413. The repair template contained 36 nucleotide homology arms flanking the loxP sequence and was synthesized as an Ultramer DNA oligo (IDT). Candidate loxP insertions were identified by co-conversion using a *dpy-10* guide and repair oligo (Arribere *et al.* 2014). All components [pSS4 *dpy-10* guide and Cas9 expression (50 ng/μl), *pbrm-1* guide plasmid (50 ng/μl), loxP repair oligo (30 ng/μl), *dpy-10* repair oligo (30 ng/μl)] were injected into RA650. F1 roller worms were placed 3 to a plate and allowed to self-fertilize. Once the food was depleted, a portion of the population was washed off the plate and treated with proteinase K to produce a crude DNA prep. These DNA preps were screened using primers in *pbrm-1* and loxP. Individual animals from populations containing a PCR product of the correct size were singled and allowed to give rise to populations with homozygous insertions; the insertion was verified by sequencing; the resulting strain is RA661. Recombination between the loxP sites would be predicted to remove the last 6 exons of *pbrm-1*, which are shared by *pbrm-1a* and *pbrm-1b,* as well as all GFP-coding exons (Fig. 1a).

### *hnd-1* promoter-driven conditional inactivation

*hnd-1p::Cre* (pRA638)*, hnd-1p::GFP-nanobody::ZIF-1::NLS-Cre* (pRA640), and *hnd-1p::ZIF-1* (pRA642) expression constructs were created in pCFJ355 (Addgene #34870), a vector designed for Mos1-mediated single-copy transgene insertion on LGX (Frokjaer-Jensen *et al.* 2008, 2012). The *hnd-1p::Cre* plasmid was generated by digesting pSR47 (Addgene #69258; Ruijtenberg and van den Heuvel 2015) with PacI and XbaI. The *hnd-1* promoter was amplified by PCR from pJK850 (Mathies *et al.* 2003) using primers RA1396 and RA1397 and inserted in place of the *myo-3* promoter in pSR47. The *hnd-1p::ZIF-1::Cre* and *hnd-1p::GFP-nanobody::ZIF-1::Cre* repair plasmids were generated by digesting pRA638 with PacI and inserting sequences upstream of Cre. ZIF-1 and GFP-nanobody::ZIF-1 sequences were amplified from pOD2046 (Addgene #89367; Wang *et al.* 2017) using primers RA1506 and RA1507 (GFP-nanobody::ZIF-1) or RA1508 and RA1507 (ZIF-1). The operon linker in pOD2046 was retained to separate the GFP-nanobody::ZIF-1 (or ZIF-1) and Cre-coding sequences, creating bicistronic expression constructs. The products were cloned into pRA638 using the NEBuilder HiFi DNA Assembly Master Mix (NEB). All primers are listed in Supplemental material online (Supplementary File 1).

Single-copy insertions were generated using a published protocol (Frokjaer-Jensen *et al.* 2008). Each Cre driver plasmid was injected into *unc-119(ed9); ttTi14024* worms at 50 ng/μl with *eft-3p::transposase* (50 ng/μl) fluorescent coinjection markers [*myo-2p::mCherry* (2.5 ng/μl), *myo-3p::mCherry* (5 ng/μl), and *sur-5p::tdTomato* (5 ng/μl)] and a negative selection marker [*HS::peel-1* (10 ng/μl)]. Resulting non-Unc worms were placed 3 to a plate and allowed to develop at 25°C until the food was nearly depleted, at which time the worms were moved to 34°C for 2 h to eliminate array-bearing animals by *peel-1* negative selection. The plates were screened about 1 week later for non-Unc animals, which are candidate insertions. Individual animals were isolated, allowed to give rise to populations, and their progeny were screened for homozygous insertions. The resulting single-copy insertions are *rdIs67 (hnd-1p::Cre)*, *rdIs74 (hnd-1p::GFP-nanobody::ZIF-1::NLS-Cre),* and *rdIs79 (hnd-1p::ZIF-1)*. Each of these transgenes was crossed with RA661 to generate strains RA663 [*pbrm-1(rd31); rdIs67*], RA678 [*pbrm-1(rd31); rdIs74*], and RA685 [*pbrm-1(rd31); rdIs79*]. RA685 was used to control for the effect of the *hnd-1* promoter, *unc-119* rescue, and ZIF-1 protein on gene expression. This allelic combination is referred to as *pbrm-1(control)*.

### Phenotypic analysis

Six first-day adult worms were placed on a plate and allowed to lay eggs for ∼6 h. Embryos or L1 larvae that remained on the plate after 48 h were scored as embryonic or L1 lethal, respectively. The L4 staged worms that developed from the embryo collections were examined for gonadogenesis defects using a dissecting microscope. Phenotypes that were classified as gonadogenesis defective were as follows: missing anterior or posterior gonad arm, disorganized gonad with a central patch of gonadal tissue, and gonad absent. Three replicates were performed for each strain, and at least 50 worms were scored for each replicate. The percentage of defects across all 3 replicates is reported in Table 1.

**Table 1.** Phenotypic effects of *pbrm-1* Cre/lox deletion.

A. Viability	Lethality (%)		
Genotype	Embryonic	Larval	*n*
*pbrm-1::GFP(flox)*	2.6	0	425
*pbrm-1::GFP(flox); hnd-1p::Cre*	2.0	0	200
*pbrm-1::GFP(flox); hnd-1p::GFPnb-ZIF-1::Cre^a^*	4.7	0	301
*pbrm-1(rd34)*	4.4	67.7	455

B. Gonadogenesis	Gonad morphology (%)			
Genotype	One arm	Abnormal	Absent	*n*
*pbrm-1::GFP(flox)*	0.2	0	0	414
*pbrm-1::GFP(flox); hnd-1p::Cre*	3.1	0	0	196
*pbrm-1::GFP(flox); hnd-1p::GFPnb-ZIF-1::Cre^a^*	12.5	0.3	0.3	287
*pbrm-1(rd34)*	20.5	4.7	3.1	127

^*a*^Also called *pbrm-1(TS-KO)*.

### RNA sample collection

Five biological replicates were performed on different days, and *pbrm-1(control)* and *pbrm-1(TS-KO)* animals were reared and collected in parallel. Populations were grown to adulthood, harvested, and treated with hypochlorite to obtain embryos. Embryos were hatched overnight in a sterile M9 medium on a rotating platform to obtain a synchronous population of early L1 larvae. Worms were washed from the plate, purified by sucrose floatation, rinsed once with M9 medium, and stored in Trizol (Ambion, Carlsbad, CA, USA) at −80°C until RNA preparation.

### RNA sequencing and analysis

RNA was isolated using the miRNeasy kit with DNase I digestion performed on the column (Qiagen, Venlo, Netherlands). All samples had RIN numbers of 9.9 or 10 when analyzed using the TapeStation System (Agilent Technologies, Santa Clara, CA, USA). RNA was polyA selected, and indexed sequencing libraries were prepared and sequenced by GeneWiz (South Plainfield, NJ, USA). The libraries were sequenced as 150-base, paired-end reads, to an average read depth of 40 million reads per sample using the Illumina HiSeq platform (Illumina, San Diego, CA, USA). The raw RNA-sequencing data were examined using FastQC (https://www.bioinformatics.babraham.ac.uk/projects/fastqc/). Sequencing reads were trimmed to remove adapter sequences and low-quality bases. Trimmed reads were mapped to the *C. elegans* genome (Ensembl genome assembly release WBcel325) using the STAR aligner version 2.5.2b (Dobin *et al.* 2013). Unique gene hit counts were calculated using feature Counts from the Subread package v.1.5.2 (Liao *et al.* 2013, 2014). Only unique reads that fell within exons were counted. Transcripts per million (TPM) values were calculated for each gene. Differential expression was determined using DESeq2 (Love *et al.* 2014). To visualize the variance among replicates and samples, principal component analysis was performed on iDEP (Ge *et al.* 2018) using regularized log-transformed data. Volcano plots were generated using R version 4.3.0. Venn diagrams were generated using BioVenn (Hulsen *et al.* 2008).

### Bioinformatics

The overrepresentation of gene ontology (GO) terms for the DEGs was determined using the statistical overrepresentation test in PANTHER (Thomas *et al.* 2003; Mi *et al.* 2013, 2017). Gene lists were compared with all genes with TPM >0 in all 5 replicates of at least one sample type using the GO Biological Process dataset and Fisher's exact test with false discovery rate (FDR) correction. The DEGs were compared with previously published gene expression datasets from sorted SGPs and hmcs (Mathies *et al.* 2019) and sorted embryonic muscle cells (Fox *et al.* 2007). Gene names were converted to WBGeneIDs using the Gene Name Sanitizer tool on WormBase, and lists were compared using the VLOOKUP function in Excel (Microsoft, Redman, WA, USA). The following subsets of *pbrm-1* DEGs were identified: (1) SGP expressed genes (FPKM ≥ 1), (2) total muscle expressed genes, (3) muscle enriched genes, (4) SGP-biased genes that had increased expression in *pbrm-1(TS-KO)* (adjusted *P* ≤ 0.05), and (5) hmc-biased genes with reduced expression in *pbrm-1(TS-KO*) (adjusted *P* ≤ 0.05; Supplementary File 2).

### Locomotion assays

Ten first-day adult worms were placed in copper rings that had been melted into the surface of agar plates. The rings served as corrals to allow for the testing of 4 strains in parallel on one plate. Worms were acclimated to the lack of food for 30 min, after which they were moved to test plates. Worm locomotion was recorded for 2 min starting at the 10-min time point. The speed of each worm was calculated using Image Pro Plus software (Media Cybernetics, Inc., Rockville, MD, USA), and an average speed for each group of worms (*n* = 1) was calculated. Six trials were performed for each genotype; all genotypes were tested simultaneously on the same plates. Two-tailed paired Student's *t*-tests were used for statistical comparisons of the basal speeds.

### Reporter validation

Six genes were selected from among the SGP-expressed *pbrm-1(TS-KO)* DEGs for reporter validation; 3 had higher expression in SGP vs hmc (SGP-biased) and 3 had higher expression in hmc vs SGP (hmc-biased) in a dataset generated from wild-type SGPs and hmcs (Mathies *et al.* 2019). Genes were prioritized based on highest fold change and lowest adjusted *P*-values, and reporters were made using PCR fusion (Hobert 2002). The pPD95.75 plasmid was modified to use the *tbb-2* 3′ UTR because it promotes high levels of expression (Dour and Nonet 2021) and lacks the background expression reported for the *unc-54* 3′ UTR (Silva-Garcia *et al.* 2019)*. tbb-2* sequence was amplified using primers RA1792 and RA1793 and cloned into pPD95.75 (Addgene plasmid #1494) digested with EcoRI and SpeI using the NEBuilder HiFi DNA Assembly Master Mix (NEB). The resulting plasmid was used as the template for the amplification of GFP and 3′ UTR sequences using primers RA1791 and RA1799. Nested forward primers (F1 and F2) and a reverse fusion primer (R) were designed for each gene. Promoter sequences were amplified from genomic DNA using the F1 and R primers and contained up to 5 kb or all sequence to the next upstream gene. The promoter and GFP 3′ UTR PCRs were combined, and a fusion product was amplified using F2 and *tbb-2* 3′ UTR primers. The PCR product was injected into N2 worms at 10–20 ng/μl with 50 ng/μl pRF4 and 50 ng/μl DNA ladder (NEB). The pRF4 plasmid produces a dominant roller phenotype; it was used as a coinjection marker (Mello *et al.* 1991). At least 2 transmitting lines were isolated for each construct. The line with the highest transmission frequency was crossed into *pbrm-1(ok843)* mutants using *oxTi718* (*eft-3p::tdTomato::H2B*) as a balancer; *oxTi718* is at 2.07 and *pbrm-1* is at 2.10 on LGI. Homozygous *pbrm-1* mutants were identified by the absence tdTomato expression, and their progenies were examined for reporter expression.

Twenty first-day adults were placed on a plate and allowed to lay eggs for 2 h. The resulting L1 larvae were examined ∼16 h later, using fluorescence and differential interference contrast (DIC) microscopy. Only L1 larvae containing 4 cells in the gonad primordium were scored because most *pbrm-1* mutants do not develop beyond this stage. GFP fluorescence in SGPs was noted, and 3 levels of expression were recorded—dim, distinct, or bright. At least 30 L1 staged worms were observed. Each SGP was assigned a ranked numerical score for the level of expression (0 = none, 1 = “dim,” 2 = “distinct,” and 3 = “bright”). Statistical comparisons were made using unpaired Student's *t*-tests in Prism version 9.5.1 (GraphPad). Reporters were visualized using a Zeiss Axioskop II microscope. All fluorescent images intended for comparison were taken with the same exposure and had identical image adjustments.

## Results

### Conditional inactivation of *pbrm-1* in mesodermal tissues

Strong loss-of-function alleles of *pbrm-1* result in a high degree of embryonic or L1 larval lethality in the progeny of homozygous animals (Large and Mathies 2014). The few escaping animals have incompletely penetrant gonadogenesis defects, indicating that there is a role for *pbrm-1* in the somatic gonad. To facilitate the analysis of this gonadogenesis function, we generated a strain that combines Cre/lox recombination with GFP nanobody-directed protein degradation to eliminate functional PBRM-1 from SGPs, while preserving it in most of the animal. First, we created a *pbrm-1* translational GFP fusion using an established CRISPR/Cas9 genome editing protocol (Dickinson *et al.* 2015). We inserted GFP following the last *pbrm-1* exon to create a C-terminal PBRM-1::GFP fusion protein. Following removal of the selection cassette, this allele retains one loxP site downstream of the GFP coding exons. We inserted a second loxP site upstream of the 10th *pbrm-1* exon, before the start of *pbrm-1b* transcription, creating the loxP-flanked allele (Fig. 1a). This reporter, hereafter called *pbrm-1::GFP(flox),* expresses GFP in most or all cells of the animal, across all life stages (Fig. 1b). The GFP insertion in *pbrm-1* has minimal effects on viability and somatic gonad development (Table 1). When recombined by Cre recombinase, the resulting deletion allele is predicted to lack the last 6 exons of *pbrm-1*, which are shared by *pbrm-1a* and *pbrm-1b,* as well as the GFP-coding sequences; it should cause a loss of function of both *pbrm-1a* and *pbrm-1b*.

**Fig. 2.**
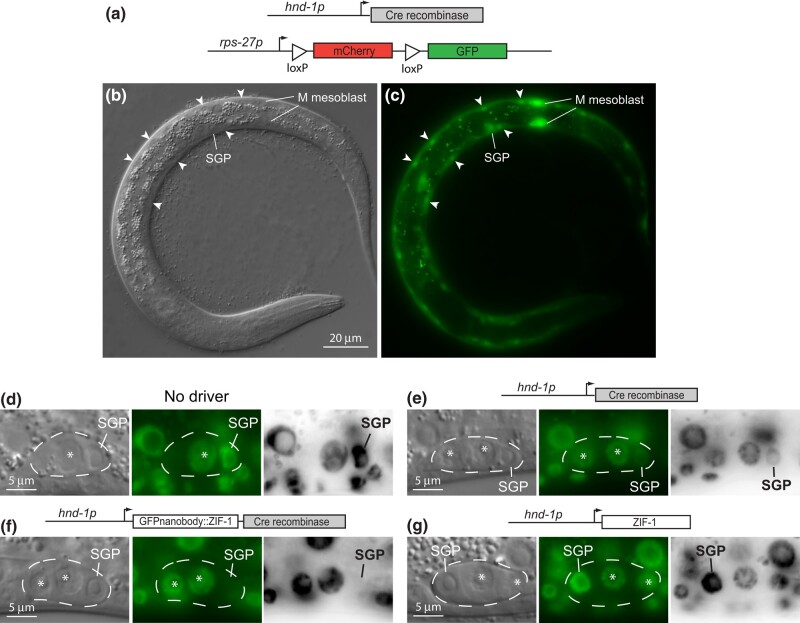
*pbrm-1* conditional knockout in SGPs. a–c) A Cre readout reporter shows where *hnd-1* promoter driven Cre recombinase is active. The readout reporter is driven by a ubiquitous promoter and switches from mCherry to GFP expression upon recombination between loxP sites. b and c) *hnd-1p::Cre* promotes recombination in SGPs, body wall muscles (arrow heads), and M mesoblast daughters; DIC image b) and GFP fluorescence c). d–g) A combination of Cre/lox recombination and GFP nanobody-directed protein degradation was used to eliminate PBRM-1::GFP from SGPs. Driver constructs are indicated above images: no driver d) *hnd-1p::Cre* e), *hnd-1p::*GFP-nanobody::ZIF-1::Cre f), *hnd-1p::*ZIF-1 alone g). DIC image (left), GFP fluorescence (middle), and inverted monochrome fluorescent image (right). The gonad primordium (dashed line) and SGPs are indicated; the asterisks mark germ cells. All fluorescent images are 1 s exposures with identical adjustments. PBRM-1::GFP fluorescence in SGPs is reduced by *hnd-1p::Cre* recombination e) and made invisible by the addition of ZIF-1–mediated degradation f); *hnd-1p::*ZIF-1 does not affect PBRM-1::GFP.

To conditionally inactivate *pbrm-1* in the SGP lineage, we used the *hnd-1* promoter to drive a nuclearly localized Cre recombinase. *hnd-1* is expressed in embryonic mesodermal tissues derived from the MS, C, and D lineages, including the grandparents of SGPs and hmcs (Mathies *et al.* 2003). We generated single-copy insertions of *hnd-1p::Cre* using Mos1-mediated single-copy insertion (MosSCI; Frokjaer-Jensen *et al.* 2008). We found that this driver resulted in Cre-mediated recombination in mesodermal cells, including body wall muscles, the M mesoblast, and SGPs, as assessed using a reporter that switches from *mCherry* to GFP expression upon Cre recombination (Ruijtenberg and van den Heuvel 2015; Fig. 2a–c). We crossed the Cre driver with *pbrm*-1*::GFP(flox)* to induce the excision of the *pbrm-1* exons and examined L1 SGPs for the expression of PBRM-1::GFP. The *hnd-1p* Cre driver substantially reduced the level of PBRM-1::GFP protein in SGPs, but it did not totally eliminate the protein (Fig. 2d and e). The residual GFP fluorescence suggested that PBRM-1 protein was produced from mRNA generated before the excision event and that perdured in SGPs. In order to remove the remaining protein, we employed a GFP nanobody fused to ZIF-1, which targets PBRM-1::GFP for ubiquitin-mediated degradation by the proteosome (DeRenzo *et al.* 2003; Wang *et al.* 2017). We created a bicistronic construct that expresses GFP-nanobody::ZIF-1 and Cre (Fig. 2f). The combination of Cre recombination and ZIF-1–mediated protein degradation eliminated all visible GFP fluorescence from L1 SGPs (Fig. 2f). To control for the effect of ZIF-1 and other genetic elements in the Cre drivers, we generated a construct expressing only ZIF-1 and confirmed that this driver did not affect PBRM-1::GFP protein levels (Fig. 2g).

To determine how the different tissue-specific inactivation strategies affect *pbrm-1* function, we examined the phenotype of each of the strains (Table 1). *pbrm-1* null or strong loss-of-function alleles result in highly penetrant embryonic or early larval lethality (Large and Mathies 2014). In contrast, all the conditional inactivation strains had minimal lethality (Table 1). Animals lacking *pbrm-1* function maternally and zygotically, and that escape lethality, have incompletely penetrant gonadogenesis defects; typically, they are missing 1 of the 2 gonad arms (Large and Mathies 2014). We found that *pbrm-1::GFP(flox)* had a low penetrance gonodogenesis defect on its own, *hnd-1p::*Cre–mediated recombination increased this penetrance to 3.1%, and the further addition of GFP nanobody-mediated protein degradation substantially increased the penetrance of the defect to 13.1% (Table 1). Therefore, by employing a combination of Cre/lox recombination and ZIF-1–mediated protein degradation, we have generated a tissue-specific (TS) *pbrm-1* knockout (KO), *pbrm-1(TS-KO),* that produces a strong loss-of-function phenotype in the somatic gonad with minimal lethality and will allow us to perform gene expression studies.

### *pbrm-1–*regulated genes

We used *pbrm-1(TS-KO)* to identify genes that are regulated by *pbrm-1* in mesodermal tissues. The strain containing the ZIF-1 driver (Fig. 2f) served as a control; these worms are *pbrm-1(+)* in all tissues, and they contain the same selectable markers as *pbrm-1(TS-KO);* we refer to this strain as *pbrm-1(control).* We previously sorted SGPs for gene expression analysis (Mathies *et al.* 2019). For this study, we chose to isolate mRNA from whole animals because *pbrm-1* loss-of-function results in SGPs that sometimes fail to express appropriate markers of their fate (Large and Mathies 2014); these SGPs might not be included in the analysis if we sorted based on expression of an SGP marker. We obtained synchronous populations of early L1 stage worms by bleaching gravid adults and allowing the embryos to hatch in the absence of food. We performed 5 replicates on different days, and we grew, collected, and processed *pbrm-1(TS-KO)* and *pbrm-1(control)* worms in parallel. RNA-sequencing libraries were prepared, sequenced, and mapped to the genome by GeneWiz (Azenta Life Sciences, Plainfield, NJ, USA).

During these experiments, we noticed that some L1 larvae from *pbrm-1(TS-KO)* lacked GFP expression entirely. Because *hnd-1* has a maternal effect (Mathies *et al.* 2003), we thought it was likely that these larvae resulted from the maternal recombination of the *pbrm-1* locus. This maternal recombination provided an opportunity to assess the effect of Cre-/lox-mediated deletion on the *pbrm-1* locus. We isolated animals lacking GFP in all tissues, which are homozygous for the *pbrm-1* deletion in the germline, and we examined their progeny for lethality and somatic gonad defects. We found that this new deletion, *pbrm-1(rd34),* had phenotypes that were seen in other strong loss-of-function *pbrm-1* alleles (Table 1). When compared with *pbrm-1(ok843)* (Large and Mathies 2014), the *rd34* allele had less lethality and a higher penetrance gonadogenesis defect. Therefore, the deletion of the C-terminal *pbrm-1* exons creates a strong loss-of-function allele that is at least as strong as the strongest reported *pbrm-1* allele in the somatic gonad. To determine the prevalence of the maternal *pbrm-1(−)* animals, we examined 3 different samples and found that 3.1% ± 1.1% of the L1 larvae had no GFP fluorescence. Since these L1 larvae are *pbrm-1* mutant in all cells, including SGPs, we reasoned that they should not significantly impact our ability to identify *pbrm-1*–regulated genes in SGPs.

**Fig. 3.**
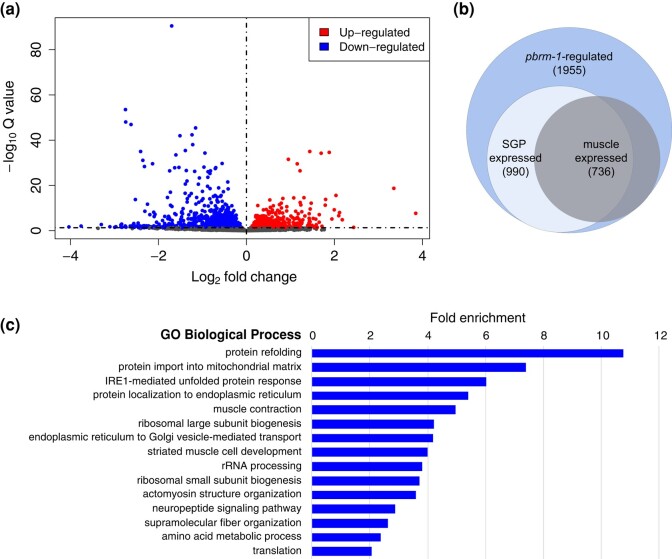
Genes regulated by *pbrm-1* in mesodermal tissues. a) Volcano plot showing the distribution of DEGs between *pbrm-1(TS-KO)* and *pbrm-1(control)*. b) Venn diagram indicating the proportion of *pbrm-1-*regulated genes that are also expressed in SGPs or embryonic muscles. The number of genes in each circle is indicated; 558 genes are expressed in both SGPs and muscles. c) GO biological process categories are statistically overrepresented among the SGP-expressed *pbrm-1-*regulated genes. Fold enrichment is plotted.

We assessed the correlation between biological replicates and found that the sample types were separated by a combination of principal components 1 and 3 (Supplementary Fig. 1). Together, these principal components account for 51% of the variance in the dataset. We examined differential gene expression using DESeq2 (Love *et al.* 2014) and found that 1,955 genes were differentially expressed between *pbrm-1(TS-KO)* and *pbrm-1(control)* (FDR ≤ 0.05; Supplementary File 2). We did not apply a fold-change cutoff to this analysis because we are interested in identifying gene expression changes that occur in 2 of the 558 cells of the newly hatched L1 larva, and we expect that some of the important changes in SGP expression may not be of large magnitude. Among the DEGs, there were similar numbers of upregulated and downregulated genes (Fig. 3a).

The *pbrm-1(TS-KO)* allele removes PBRM-1 from SGPs and other mesodermal cells, primarily body wall muscle (Mathies *et al.* 2003). Therefore, to enrich for genes that function in SGPs, we first filtered the DEGs against all genes that were expressed in sorted SGPs (Mathies *et al.* 2019). This resulted in 990 candidate *pbrm-1*–regulated genes in SGPs (Fig. 3b; Supplementary File 2). We examined the genes for GO biological process terms and found 2 overrepresented categories related to muscle function, “muscle contraction” and “striated muscle cell development” (Fig. 3c; Supplementary File 3). The identification of muscle GO terms suggested that the filter for SGP-expressed genes did not eliminate those that also function in muscles. Consistent with this observation, we found that 558 of the 990 *pbrm-1*–regulated genes expressed in SGPs were also expressed in embryonic muscle cells (Fig. 3b; Supplementary File 2; Fox *et al.* 2007). Most of the genes in the muscle GO categories were enriched in embryonic muscle cells (Table 2), and nearly all were downregulated in *pbrm-1(TS-KO),* suggesting that *pbrm-1* promotes muscle differentiation by regulating genes that are essential for muscle function.

**Table 2.** Muscle function genes overrepresented among *pbrm-1–*regulated genes.

Gene	Description	Muscle enriched	*pbrm-1* expression
*dyb-1*	Alpha-dystrobrevin	No	Down
*lev-11*	Tropomyosin	Yes	Down
*mel-26*	BTB protein	No	Up
*mup-2*	Troponin T	Yes	Down
*myo-6*	Myosin heavy chain	Yes	Down
*pat-10*	Troponin C	Yes	Down
*pat-3*	Beta-integrin	Yes	Down
*tni-3*	Troponin I	Yes	Down
*tnt-2*	Troponin T	Yes	Down
*tnt-4*	Troponin T	No	Down
*unc-27*	Troponin I	Yes	Down
*unc-52*	Perlecan	Yes	Down

Since *pbrm-1* regulates the expression of genes required for muscle contraction, we reasoned that we might see defects in locomotion in *pbrm-1(TS-KO)* worms. Locomotion is a neuromuscular process, and mutations affecting muscle function result in uncoordinated movement and reduced locomotion speed (Gieseler *et al.* 2017). Wild-type worms travel at ∼200 μm/s in the absence of food in these assays (Table 3). The *pbrm-1* translational GFP insertion did not significantly alter locomotion speed, while both *pbrm-1(TS-KO)* and *pbrm-1(control)* exhibited slower locomotion speeds. Importantly, there was no significant difference in speed between *pbrm-1(TS-KO)* and *pbrm-1(control)*, suggesting that *pbrm-1* is not required for normal locomotion. One caveat to the interpretation of these results is that both strains are homozygous for a loss-of-function *unc-119* allele and carry 2 wild-type copies of the *C. briggsae unc-119* gene (Frokjaer-Jensen *et al.* 2008). Since *unc-119* mutants have severely reduced locomotion speed (Maduro and Pilgrim 1995), we hypothesize that incomplete rescue of the *unc-119* mutation causes the reduced locomotion speed seen in *pbrm-1(TS-KO)* and *pbrm-1(control)*. If this is the case, it might also mask any subtle effect of *pbrm-1* on locomotion.

**Table 3.** Locomotion speed of pbrm-1 strains.

Genotype	Speed (µm/s)*^a^*	*P*-value
Wild type	205.9 ± 3.3	
*pbrm-1::GFP(flox)*	199.5 ± 5.6	0.30*^b^*
*pbrm-1(TS-KO)*	137.8 ± 9.5	0.0023*^c^*, 0.15*^d^*
*pbrm-1(control)*	111.8 ± 7.1	0.0004*^c^*

^*a*^Average speed ± SEM.

^*b*^Compared with wild type.

^*c*^Compared with *pbrm-1::GFP(flox)*.

^*d*^Compared with *pbrm-1(control)*.

One other intriguing overrepresented GO category is “neuropeptide signaling pathway.” Almost all the *pbrm-1*–regulated genes in this category encode neuropeptide-like proteins or FMRF-like peptides. We previously showed that the sister of the SGPs, the hmc, expresses many secretory proteins, including over 30 FMRF-like peptides (Mathies *et al.* 2019). Because *pbrm-1* is important for the cell fate decision that distinguishes SGPs from their hmc sisters (Large and Mathies 2014), one simple model is that neuropeptide signaling genes are upregulated in *pbrm-1* mutant SGPs because they are transformed toward the hmc fate. We examined the *pbrm-1*–regulated neuropeptide signaling genes in the SGP/hmc expression dataset and found that 6 of 22 were normally expressed at higher levels in hmc than in SGPs (hmc-biased), and all of these hmc-biased genes had increased expression in *pbrm-1* mutants (Supplementary File 3). These genes are excellent candidates for hmc differentiation genes that are upregulated in SGPs because of the transformation of SGPs to hmcs in *pbrm-1* mutants.

As a first validation step for this dataset, we sought to confirm that *pbrm-1* expression was reduced in *pbrm-1(TS-KO)* when compared with *pbrm-1(control)*. The differential expression analysis was performed using gene-based counts, and in this analysis, we did not identify *pbrm-1* as a DEG. Since Cre-mediated recombination of the *pbrm-1* locus only removes exons 10–15 (Fig. 1a), we might not expect to detect differential expression using gene-based counts. We performed differential gene expression analysis at the exon level using DEXSeq (Li *et al.* 2015) and found that 5 of the last 6 exons had a statistically significant reduction in expression in *pbrm-1(TS-KO)* compared with *pbrm-1(control)* (Supplementary Fig. 1 and File 4). We conclude that we can detect *pbrm-1* mRNA expression differences that are restricted to mesodermal tissues in RNA isolated from whole L1 animals.

### *pbrm-1–*regulated genes in SGPs

Our goal with the tissue-specific knockout of *pbrm-1* was to identify genes that are targets of *pbrm-1* in the SGP/hmc fate decision. We are particularly interested in 2 groups of genes. First are genes that are positively regulated by *pbrm-1* and normally expressed at higher levels in SGPs than in hmc (SGP-biased); we identified 125 genes in this class (Supplementary File 2); these are candidate positive regulators of the fate and multipotency of SGPs. Second are genes that are negatively regulated by *pbrm-1* and are normally expressed at higher levels in hmc than in SGPs (hmc-biased); we identified 53 genes in this class (Supplementary File 2); these are candidate hmc differentiation genes or negative regulators of multipotency that are repressed in SGPs. We selected 3 genes in each category for reporter validation: *hsp-4, dpy-18,* and *txdc-12.1* are normally SGP biased and are downregulated in *pbrm-1(TS-KO)*, while *hsp-12.3, acdh-1,* and *nlp-58* are normally hmc biased and are upregulated in *pbrm-1(TS-KO)*. We generated transcriptional reporters of each gene and examined them for expression in SGPs or hmc. All of the reporters had some expression in L1 larvae. Of the SGP-biased gene reporters, *dpy-18::GFP* and *txdc-12.1::GFP*, had detectable expression in SGPs, while *hsp-4::GFP* did not. Of the hmc-biased genes, only *hsp-12.3::GFP* had detectable expression in hmc.

**Fig. 4.**
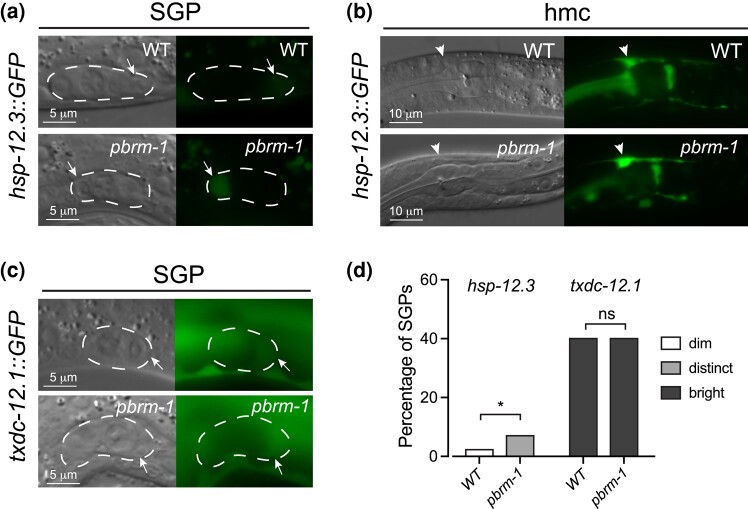
Genes regulated by *pbrm-1* in SGPs. Expression of reporters in wild-type (WT) and *pbrm-1(ok843)* mutants. Paired images are DIC (left) and GFP fluorescence (right). Arrows point to SGPs; arrowheads point to hmc; the gonad primordium is outlined (dashed line). Scale bars are indicated. *hsp-12.3::GFP* expression in SGP a) and hmc b); fluorescent exposures are 500 ms. a) *hsp-12.3::GFP* is expressed very faintly in wild type SGPs (top), and this expression increases in *pbrm-1(ok843)* mutants (bottom). b) *hsp-12.3::GFP* is expressed in hmc (top); this expression is unchanged in *pbrm-1(ok843)* mutants (bottom). c) *txdc-12.1::GFP* is expressed in WT SGPs (top), and this expression is unchanged in *pbrm-1(ok843)* mutants (bottom); fluorescent exposures are 50 ms. Paired WT and *pbrm-1* mutant images were taken with identical exposures and adjustments for comparison. d) Percentage of SGPs with dim (white), distinct (light grey), or bright (dark grey) GFP in SGPs. Unpaired Student's *t*-tests were used to compare expression in WT and *pbrm-1* mutants; *P ≤ 0.05.

We chose *hsp-12.3::GFP* and *txdc-12.1::GFP* for further analysis because they had more limited expression in cells other than SGPs and hmc. We crossed each reporter into *pbrm-1(ok843)* mutants and examined their expression patterns in the wild-type and mutant backgrounds. The *hsp-12.3* reporter was expressed in hmc and, only occasionally and very weakly, in SGPs (Fig. 4a and b). In *pbrm-1* mutants, *hsp-12.3::GFP* was expressed more frequently and at higher levels in SGPs (Fig. 4a and d), suggesting that *hsp-12.3* is normally repressed by *pbrm-1* in SGPs. The *txdc-12.1* reporter was expressed in SGPs, and it had similar expression in wild-type and *pbrm-1* mutants (Fig. 4b and d), suggesting that either *txdc-12.1* is not regulated by *pbrm-1* in SGPs or the transcriptional reporter does not include all of the sequences required for proper regulation. Importantly, the reporter analysis identified one target of *pbrm-1* that could play a role in the SGP/hmc cell fate decision, indicating that this dataset can be used to identify these genes.

## Discussion

*pbrm-1* encodes the signature subunit of the PBAF complex, which is important for distinguishing multipotent SGPs from their differentiated sister cell, the hmc (Large and Mathies 2014). We used a tissue-specific gene inactivation strategy to identify genes that are regulated by *pbrm-1* in SGPs. Our approach used Cre/lox recombination to remove *pbrm-1* exons that are common to all PBRM-1 isoforms and GFP nanobody-targeted protein degradation to remove residual protein. This combination produced a strong loss-of-function phenotype in the somatic gonad and the absence of any visible PBRM-1::GFP in SGPs, strongly suggesting that PBRM-1 targets in SGPs are among the DEGs in *pbrm-1(TS-KO)*.

Cre/lox recombination of the floxed *pbrm-1* allele results in the deletion of PBRM-1B and truncation of PBRM-1A after amino acid 1346, removing the DNA-binding HMG domain. The strongest existing *pbrm-1* allele by phenotypic and molecular criteria is *ok843,* which truncates PBRM-1A after amino acid 390 and leaves PBRM-1B unaffected (Fig. 1a; Large and Mathies 2014). Animals carrying the Cre/lox deletion, *rd34*, in their germline had phenotypes similar to *pbrm-1(ok843)*, including embryonic and larval lethality and missing gonadal arms, indicating that this deletion causes a strong loss of gene function. The *rd34* allele resulted in less lethality and a higher penetrance gonadogenesis defect than *ok843*, suggesting that different PBRM-1 domains may be important for these 2 developmental functions of the gene. The stronger effect of *pbrm-1(rd34)* on gonadogenesis could point to important functions for PBRM-1B in the somatic gonad. Conversely, the stronger effect of *pbrm-1(ok843)* on viability suggests that protein domains removed by this deletion may be important for embryogenesis or early larval development.

Cre/lox deletion of the last 6 exons of *pbrm-1* produced a phenotype that resembled zygotic loss of function for strong *pbrm-1* deletion alleles in the somatic gonad. *pbrm-1* alleles exhibit maternal effects on somatic gonad development (Large and Mathies 2014), which can be explained by the inheritance of maternal PBRM-1 protein by SGPs. Consistent with this, the addition of PBRM-1 degradation in the mesoderm produced a phenotype that resembled maternal and zygotic loss of *pbrm-1* function in the somatic gonad. These observations are consistent with a previous study that showed dose-dependent functions of the SWI/SNF complex in the regulation of cell division in the M mesoblast: the incomplete loss of *swsn-1* via Cre/lox recombination or GFP nanobody-directed protein degradation produced an overproliferation phenotype, while the combination of both produced an underproliferation phenotype (van der Vaart *et al.* 2020). Together, these 2 studies argue strongly for the use of both genetic deletion and protein degradation to produce a strong loss of gene function in specific tissues.

### *pbrm-1–*regulated genes in the SGP/hmc fate decision

To understand how *pbrm-1* influences the SGP/hmc fate decision, we sought to identify genes whose regulation and expression suggested a role in this cell fate decision. We identified genes in 2 categories: (1) those that were positively regulated by *pbrm-1* and normally SGP biased (125 genes) and (2) those that were negatively regulated by *pbrm-1* and normally hmc biased (53 genes). We used fluorescent reporters to examine the expression and regulation of 3 genes in each category. Of the 6 reporters, 3 had the expected expression in SGPs or hmc. This is consistent with our previous work, in which we found that 2 of 5 genes with expression in sorted SGPs were validated by reporters (Mathies *et al.* 2019). There are many reasons that traditional transgenic reporters may not accurately reflect the expression of the gene, the most significant of which is that they do not contain all relevant regulatory sequences. Indeed, in our previous work, we showed that when we used CRISPR/Cas9 genome editing to make an endogenous reporter for a gene that did not mirror the RNA sequencing results, we observed the expected expression pattern, strongly supporting the notion that transcriptional reporters may not always accurately recapitulate the endogenous expression pattern.

We further examined the regulation of reporters for *hsp-12.3* and *txdc-12.1. hsp-12.3* was upregulated in *pbrm-1(TS-KO)*, and consistent with this, we found that the reporter was more highly expressed in SGPs in *pbrm-1* mutants, indicating that *hsp-12.3* is normally repressed by *pbrm-1* in SGPs. *txdc-12.1* was downregulated in *pbrm-1(TS-KO)*, but the reporter did not show any change in expression in *pbrm-1* mutant SGPs. One explanation for this result is that *txdc-12.1* is not regulated by *pbrm-1* in SGPs; instead, it may be regulated by *pbrm-1* in other mesodermal tissues. Alternatively, the reporter might be missing the regulatory sequences that mediate regulation by *pbrm-1* in SGPs. Ultimately, it will be necessary to examine reporters in the native genomic context to fully characterize the regulation of these genes by *pbrm-1*.

Our tissue-specific inactivation strategy provides a significant improvement over gene expression studies using conventional germline mutations, which affect all cells in the animal and that result in a high degree of lethality. However, this approach does have some limitations for identifying *pbrm-1*–regulated genes in SGPs. First, to inactivate *pbrm-1* early in the SGP lineage, we also had to inactivate it in other mesodermal tissues. The *hnd-1* promoter is expressed in the grandparents of SGPs (and hmcs) and again shortly after the SGPs are born (Mathies *et al.* 2003). We chose this promoter because it could be used to inactive *pbrm-1* prior to the SGP/hmc fate decision. The *hnd-1* promoter also drives expression earlier in the MS, C, and D lineages, which produce a total of 83 cells including 70 body muscles, 2 SGPs, the hmc, and 10 other mesodermally derived cells (Sulston *et al.* 1983). We therefore expect the dataset to include *pbrm-1*–regulated genes in each of these cell types. Since there are many more muscle cells than SGPs, we anticipated that we would identify *pbrm-1*–regulated genes in muscles, and indeed, GO terms related to muscle function were an overrepresented category in the DEGs. Second, the *hnd-1*–driven Cre recombinase causes a low level of maternal recombination resulting in animals that are mutant for *pbrm-1* in all 558 cells of the L1 larva, including SGPs. Both limitations will reduce the sensitivity to detect *pbrm-1*–regulated genes in SGPs. Nonetheless, we identified 178 candidate genes and confirmed one gene, *hsp-12.3,* that is regulated by *pbrm-1* in SGPs.

### Muscle differentiation genes are positively regulated by *pbrm-1*

*hnd-*1–driven gene inactivation eliminates PBRM-1 protein from most of the body wall muscles. We found that genes with GO terms related to muscle function were overrepresented among the *pbrm-1*–regulated genes in this dataset. The *C. elegans* body wall musculature differentiates in embryogenesis just prior to the 2-fold stage (Hresko *et al.* 1994). Our gene expression analysis was performed on L1 stage larvae, in which muscles are fully differentiated. We found 12 DEGs related to muscle contraction or striated muscle development, and all but one of these genes had reduced expression in *pbrm-1(TS-KO),* indicating that *pbrm-1* normally promotes the expression of these genes.

We predicted that the loss of muscle gene expression might affect worm locomotion. However, we did not observe any difference in locomotion speed between *pbrm-1(−)* and *pbrm-1(+)* worms, suggesting that *pbrm-1* is not required in muscles for normal locomotion. One possibility is that the gene expression changes were not significant enough to impact muscle structure and therefore affect locomotion. Most of the muscle genes had modestly reduced expression, ranging from 70% to 90% of the levels in *pbrm-1(+)* (Supplementary File 3). This analysis was complicated by the fact that both strains used for the RNA-Seq analysis had reduced locomotion speed compared with wild type. These strains carry single-copy insertions that were generated using the MosSCI technique, which uses the rescue of an *unc-119* mutant phenotype as a selectable marker (Frokjaer-Jensen *et al.* 2008). *unc-119* mutants have significantly reduced locomotion speed (Maduro and Pilgrim 1995). Therefore, it is likely that the reduced locomotion speed in these strains is due to the incomplete rescue of the *unc-119* phenotype, and it is possible that the locomotion defect may be masking any subtle effect of mesodermal *pbrm-1* inactivation on locomotion.

Mammalian SWI/SNF complexes promote MyoD-dependent muscle differentiation, and about a third of MyoD-induced genes require SWI/SNF function (de la Serna *et al.* 2001; de la Serna *et al.* 2005). These studies specifically implicated the core ATPase subunits of SWI/SNF, Brahma (Brm) and Brahma-related gene-1 (Brg1). *C. elegans* has a single gene, *swsn-4,* encoding the SWI/SNF ATPase subunit (Sawa *et al.* 2000). Based on phenotypic analyses, it is predicted that SWSN-4 is incorporated into both BAF and PBAF complexes (Shibata *et al.* 2012; Large and Mathies 2014). The finding that PBRM-1 regulates the expression of muscle differentiation genes in *C. elegans* raises the possibility that the PBAF complex promotes muscle differentiation across phyla. It further suggests that this dataset may provide insight into how PBAF regulates the differentiation of muscles.

## Supplementary Material

jkad297_Supplementary_Data

## Data Availability

Strains are available upon request. The RNA sequencing dataset generated during this study is available in the NCBI Sequence Read Archive (SRA), accession number PRJNA1027254, and raw counts are available in the NCBI Gene Expression Omnibus (GEO), accession number GSE249603. Supplemental material available at G3 online.
