# Supplementary material for: PBRM-1/PBAF-regulated genes in a multipotent progenitor in Caenorhabditis elegans
Source: G3 (Bethesda). 2023 Dec 27;14(3):jkad297. doi: 10.1093/g3journal/jkad297 (PMC10917506; doi:10.1093/g3journal/jkad297)
Supplement: jkad297_Supplementary_Data [file jkad297_supplementary_data.zip › Supplemental_Material_G3-2023-404630.docx]

**Figure S1. RNA sequencing analysis.** (A) Principal component analysis. Gene expression profiles plotted against principal components one and three (PC1 and PC3). *pbrm-1(TS-KO)* and *pbrm-1(control)* replicates group separately. (B) Exon-level differential gene expression analysis for *pbrm-1.* Expression level in *pbrm‑1(TS-KO)* (blue) or *pbrm-1(control)* (red) is plotted for each exon. Significant differences are indicated by pink exons on the gene diagram. Exons deleted by Cre/lox recombination in the *pbrm-1(TS-KO)* allele are indicated. Results of the DEXSeq analysis are in File S4. E006, E011, and E019 do not correspond to known exons and are only 2 or 3 bps in length.
