# Supplementary figures and images for: PBRM-1/PBAF-regulated genes in a multipotent progenitor in Caenorhabditis elegans
Source: G3 (Bethesda). 2023 Dec 27;14(3):jkad297. doi: 10.1093/g3journal/jkad297 (PMC10917506; doi:10.1093/g3journal/jkad297)

Figure S1

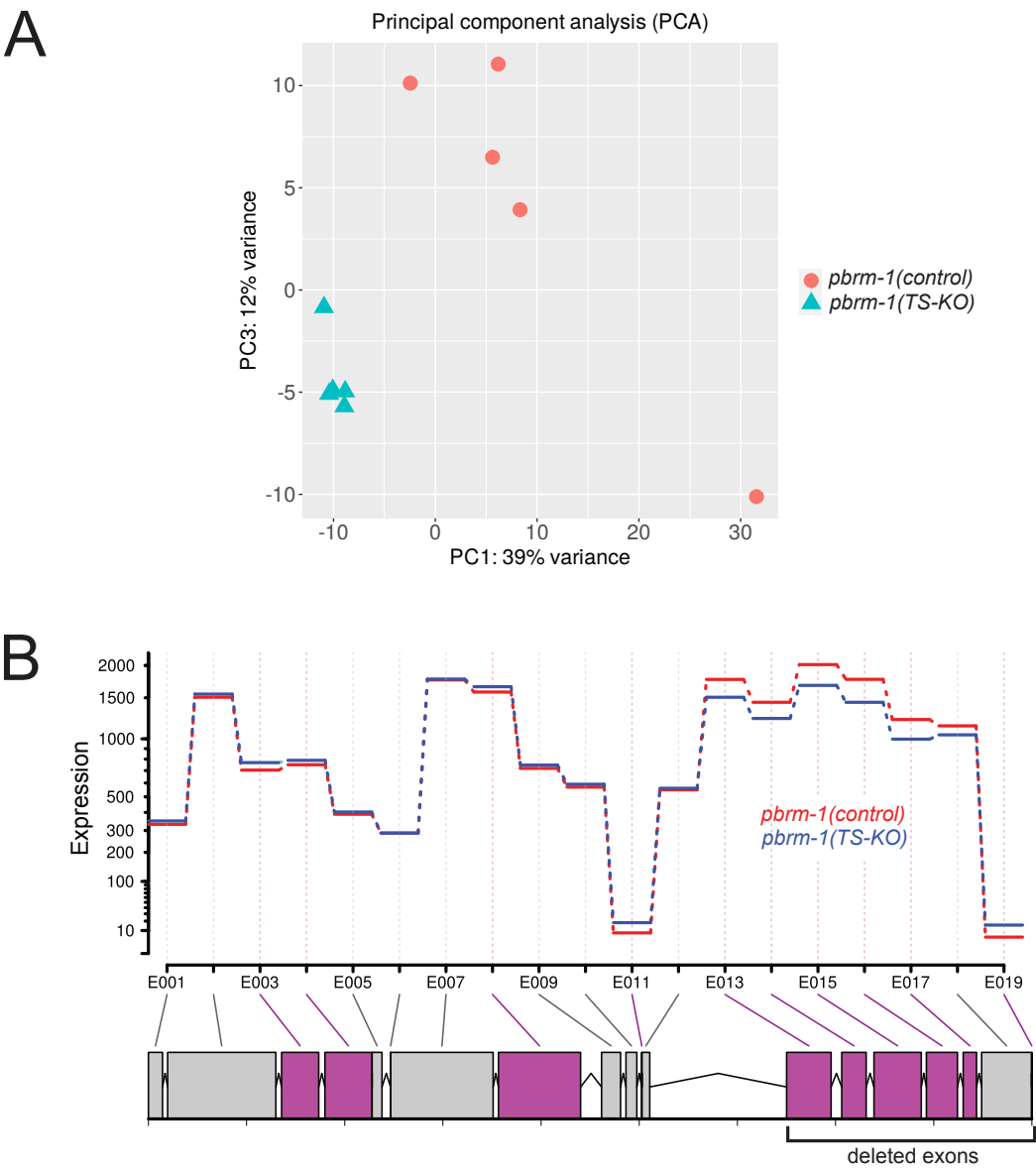

Supplement: jkad297_Supplementary_Data [file jkad297_supplementary_data.zip › Figure_S1_G3-2023-404630.pdf]
